# Functional and Blood-Based Biomarkers of Brain Aging in Senior Airline Pilots Approaching Mandatory Retirement: A Case-Control Comparison With Age-Matched Office Workers

**DOI:** 10.7759/cureus.90822

**Published:** 2025-08-23

**Authors:** Piercarlo Minoretti, Giovanni Fortuna, Konstantinos Lavdas, Davide D’Acquino

**Affiliations:** 1 Occupational Health, Studio Minoretti, Oggiono, ITA

**Keywords:** age, airline pilots, biochemical markers, brain aging, cognitive markers, retirement

## Abstract

Background

While mandatory retirement ages for commercial air transport (CAT) pilots are based on assumptions about age-related cognitive decline, empirical evidence examining brain aging in senior professionals in this field remains scarce. The aim of this study was to compare functional and blood-based biomarkers of brain aging between senior CAT pilots approaching retirement and age-matched office workers.

Methods

We conducted a case-control study involving 31 male CAT pilots aged 60-64 years and 31 age- and sex-matched office workers. Following the Aging Biomarker Consortium framework, we assessed functional biomarkers of brain aging, including processing speed (Trail Making Test Part A), episodic memory (Cued Recall Test and Auditory Verbal Learning Test [AVLT]), and fine motor control (Nine-Hole Pegboard Test). In addition, plasma concentrations of phosphorylated tau-181 (p-tau181) and neurofilament light chain (NfL) were measured as blood-based biomarkers of brain aging.

Results

Senior CAT pilots demonstrated significantly superior processing speed (Trail Making Test Part A: 26.8 ± 4.1 *versus* 31.5 ± 5.0 seconds; *p* < 0.001) and episodic memory performance (cued recall: 13.2 ± 1.9 *versus* 11.8 ± 2.3; p = 0.007; AVLT: 56.7 ± 7.8 *versus* 51.4 ± 8.1; p = 0.002) compared to office workers. However, no differences were observed in fine motor control. Regarding biochemical markers, CAT pilots showed significantly lower plasma NfL concentrations compared to office workers (7.5 ± 2.4 *versus* 9.9 ± 3.5 pg/mL; p = 0.012), whereas p-tau181 levels did not differ between groups. Across the entire cohort, higher plasma NfL concentrations were associated with slower processing speed (r = -0.30, p = 0.001), an association that remained significant after multivariable adjustment (standardized β = -0.27, p = 0.003).

Conclusions

Our findings suggest that experienced CAT pilots exhibit cognitive performances and a biochemical marker profile associated with healthier brain aging, raising questions about assumptions underlying mandatory retirement policies based solely on age, though the mechanisms underlying these differences warrant further investigation.

## Introduction

Pilot performance in commercial air transport (CAT) is crucial for aviation safety and for keeping public trust in air travel [1, 2]. As Western countries experience demographic shifts characterized by low fertility and low mortality rates [3], and considering that the industry faces a looming CAT pilot shortage - with 2023 forecasts indicating that approximately 50% of the commercial airline workforce will retire within the next 15 years [4] - stakeholders are increasingly questioning the adequacy of current mandatory retirement age limits. At present, the European Aviation Safety Agency (EASA) requires CAT pilots to retire at age 60 for single-pilot operations and at age 65 for multi-pilot roles. Similarly, the United States, under the Federal Aviation Administration Reauthorization Act of 2024, mandates a retirement age of 65 for CAT pilots, in line with International Civil Aviation Organization (ICAO) standards [4]. The establishment of current age limits is justified by concerns that age-related declines in cognitive functions requisite for safe aviation - such as processing speed, memory, and executive control - could compromise flight safety; accordingly, this risk is substantiated by evidence from both longitudinal [5] and cross-sectional, task-based research [6]. In addition, there has been evidence documenting age-related changes in piloting performance, including increased fatigue susceptibility and reduced ability to adapt to new technologies or operational procedures [4]. At the same time, potential confounders - such as overall health status, physical activity, and sleep quality - may also impact performance and should be considered when interpreting age-related differences [4]. The debate over mandatory retirement, however, involves complex considerations. In this context, the extensive professional experience accumulated over a CAT pilot's career may help offset age-related declines in cognitive performance, as senior pilots often draw on advanced judgment, knowledge, and decision-making skills to maintain high standards of safety and operational effectiveness [5, 6]. Given these complexities, a more rigorous, evidence-based foundation for aviation policy is required. A key obstacle, however, is the scarcity of empirical research that directly assesses biomarkers of brain aging in senior CAT pilots who are approaching the current retirement age. Targeted longitudinal studies with repeated assessments of functional performance, complemented by multimodal designs integrating neuropsychological testing and neuroimaging, would be ideal to directly address this gap.

Recent initiatives by the Aging Biomarker Consortium have culminated in a consensus statement that categorizes biomarkers capable of capturing multidimensional and multiscale brain changes during aging and highlights those with clinical applicability [7]. Within this framework, several functional biomarkers - including processing speed, episodic memory, and fine motor control - have been identified as having particularly strong evidence bases and recommendations for clinical use [7]. Beyond functional measures, the consortium also recognized moderate evidence supporting the use of blood-based biomarkers of brain aging [7] - including plasma phosphorylated tau at threonine 181 (p-tau181) [8] and non-disease-specific indicators of neuroaxonal injury, including neurofilament light chain (NfL) [9].

In this study, we hypothesized that senior CAT pilots would exhibit a neurocognitive and biochemical profile indicative of healthier brain aging compared to their non-pilot counterparts. Therefore, our primary objectives were 1) to compare validated functional biomarkers of brain aging - specifically processing speed, episodic memory, and fine motor control - between senior CAT pilots and an age- and sex-matched control group of office workers; 2) to compare key blood-based biomarkers of brain aging, including plasma NfL and phosphorylated tau-181 (p-tau181), between the two groups; and 3) to investigate the relationship between these functional and biological markers across the entire study cohort.

## Materials and methods

Participants

This case-control study included 31 senior CAT pilots (all male, aged 60-64 years) and a control group of 31 office workers. All pilot participants were Caucasian, employed by major airlines operating under EASA and FAA regulations, and held current Class 1 medical certification for commercial flight duties. Due to their age, all pilots occupied multi-crew positions. Participants were enrolled during routine occupational health evaluations at outpatient clinics (Studio Minoretti, Oggiono, Italy), with invitations to participate extended by an occupational health physician. Female pilots were not included due to their limited representation in the eligible sample. Office workers were recruited from administrative roles and matched to airline pilots by age and sex. Exclusion criteria comprised a history of major diseases and current or recent (within the past 90 days) medication use. None of the participants were taking dietary supplements, and all were deemed to be in good physical health. Age, sex, body mass index (BMI), years of education, and blood pressure values were recorded for all participants. The study protocol was approved by the local ethics committee (Studio Minoretti; reference number: 2023/APBA), and written informed consent was obtained from each participant in accordance with the Declaration of Helsinki.

Functional brain aging markers

All examiners were trained in standardized administration and scoring procedures and were blinded to participant group assignment to minimize assessment bias. To assess processing speed, participants completed the Trail Making Test Part A [10]. In this task, individuals were instructed to connect a series of 25 numbered circles, distributed randomly on a sheet, in ascending order as rapidly and accurately as possible. The examiner began timing at the participant’s first movement and continued timing until the final circle was connected; the total completion time, recorded in seconds, served as the main outcome and was used as a measure of processing speed and visual scanning abilities. If an error occurred, such as connecting circles out of sequence or skipping a number, the examiner immediately pointed out the mistake. The participant was required to correct the error before continuing, but the timer was not stopped during this process. Both the completion time and the number of errors were documented, with longer times or increased errors interpreted as evidence of impaired processing speed. Episodic memory was evaluated utilizing two complementary methods. In the cued recall test [11], participants were first presented with word lists to learn, after which retrieval was prompted using specific category cues corresponding to each word. Scoring was based on the number of correctly recalled words in response to these cues, reflecting retrieval accuracy and associative memory processes. Higher scores indicated greater recall ability, while lower scores suggested possible memory deficits. The cued recall test has established construct validity as a measure of episodic memory and demonstrates acceptable test-retest reliability in older adult samples [11], supporting its use in clinical and research settings. As a second measure, the Auditory Verbal Learning Test (AVLT) [12] was administered, requiring subjects to listen to and immediately recall a list of unrelated words across multiple learning trials. After the initial trials, an interference list was presented to assess susceptibility to interference, followed by immediate and delayed recall trials, and finally, a recognition trial in which participants were asked to identify previously learned words among distractors. Scoring for the AVLT included the total number of words correctly recalled on each trial, retention scores (e.g., words recalled after a delay), the degree of interference observed, and recognition hits, providing a comprehensive profile of learning capacity, retention, and susceptibility to distraction. Fine motor skills were evaluated using the nine-hole pegboard test [13]. In this task, participants were instructed to insert nine pegs, one at a time, into nine holes on a board and then remove them as quickly as possible, first using one hand and then the other. The time taken to complete the placement and removal for each hand was recorded in seconds, providing an objective measure of finger dexterity and hand-eye coordination; shorter completion times reflected better fine motor performance.

Biochemical brain aging markers

Venous blood samples were collected from participants between 8:00 and 9:00 a.m. following an overnight fast. Plasma was separated within 2 hours of collection by centrifugation at 1,300 × g for 10 minutes at 4 °C. The supernatant was then aliquoted and stored at -80 °C until analysis. Plasma concentrations of p-tau181 and NfL were measured using enzyme-linked immunosorbent assay kits (MyBioSource, San Diego, CA, USA), following the manufacturer’s protocols. The assay sensitivities were 1.0 pg/mL for p-tau181 and 7.8 pg/mL for NfL. All samples were measured in duplicate, and the technician performing the analyses was blinded to the participants’ professional information. Both inter- and intra-assay coefficients of variation were below 9.0%.

Statistics

All calculations were conducted using SPSS version 20.0 (IBM Corp., Armonk, NY, USA). The normality of data distribution was assessed using the Kolmogorov-Smirnov test. Normally distributed continuous variables are presented as means and standard deviations (SDs). Between-group comparisons for senior CAT pilots and age-matched office workers were performed using independent samples Student’s t-tests for continuous variables and chi-square tests for categorical variables. Associations between study variables were examined using Pearson's correlation coefficients and multiple linear regression; the latter included age, years of education, BMI, and systolic/diastolic blood pressure as covariates. Statistical significance was set at p < 0.05 (two-tailed) for all analyses.

## Results

General characteristics of the study participants

All participants completed the assessment battery for functional brain aging markers and provided analyzable plasma samples for biochemical brain aging markers. No significant differences were observed between senior CAT pilots and office workers regarding age, sex distribution, years of education, BMI, or blood pressure values (Table 1).

**Table 1:** Characteristics of senior airline pilots and office workers Abbreviations: SD, standard deviation; BMI, body mass index; BP, blood pressure. Values represent mean ± SD or number (percentage), as appropriate. For continuous variables, test statistics are from independent samples t-tests; for categorical variables, chi-square tests were used.

Characteristic	Pilots (n = 31)	Office workers (n = 31)	Test statistic	p-value
Age, years (mean ± SD)	62.1 ± 1.4	62.2 ± 1.5	t = 0.14	0.89
Male sex, n (%)	31 (100%)	31 (100%)	χ² = 0.00	> 0.99
Years of education (mean ± SD)	16.3 ± 2.1	15.9 ± 2.5	t = 0.83	0.41
BMI, kg/m^2^ (mean ± SD)	25.7 ± 2.8	26.1 ± 3.2	t = 0.97	0.34
Systolic BP, mm Hg (mean ± SD)	128 ± 12	130 ± 13	t = 0.88	0.38
Diastolic BP, mm Hg (mean ± SD)	79 ± 9	80 ± 8	t = 0.57	0.57

Functional brain aging markers

Senior CAT pilots exhibited significantly superior performance in processing speed and episodic memory compared to age-matched office workers (Table 2). Specifically, pilots completed the Trail Making Test Part A significantly faster than controls (mean [SD]: 26.8 [4.1] versus 31.5 [5.0] seconds; t = 4.09, p < 0.001). Pilots also achieved higher episodic memory scores, with superior mean [SD] cued recall (13.2 [1.9] versus 11.8 [2.3]; t = 2.83, p = 0.007) and greater total learning on the Auditory Verbal Learning Test (AVLT; 56.7 [7.8] versus 51.4 [8.1]; t = 3.31, p = 0.002). In contrast, there were no significant group differences in fine motor performance, as measured by the Nine-Hole Pegboard Test for either hand.

**Table 2:** Cognitive and motor performance in senior airline pilots and office workers Abbreviations: AVBL, auditory verbal learning test; SD, standard deviation. Test statistics for continuous variables are from independent samples t-tests.

Measure	Pilots (n = 31)	Office workers (n = 31)	Test statistic	p-value
Trail making test part A (sec, mean ± SD)	26.8 ± 4.1	31.5 ± 5.0	t = 4.09	<0.001
Cued recall test (score, mean ± SD)	13.2 ± 1.9	11.8 ± 2.3	t = 2.83	0.007
AVLT total learning (score, mean ± SD)	56.7 ± 7.8	51.4 ± 8.1	t = 3.31	0.002
Nine-hole pegboard, right hand (sec, mean ± SD)	20.1 ± 2.2	20.5 ± 2.3	t = 0.69	0.49
Nine-hole pegboard, left hand (sec, mean ± SD)	21.3 ± 2.5	21.7 ± 2.7	t = 0.65	0.52

Biochemical brain aging markers

There were no significant differences between senior CAT pilots and office workers in plasma levels of p-tau181; (mean [SD]: 1.36 [0.07] vs. 1.39 [0.08] pg/mL; t = 1.36, p = 0.18). In contrast, pilots showed significantly lower plasma NfL concentrations compared to controls (mean [SD]: 7.5 [2.4] vs. 9.9 [3.5] pg/mL; t = 2.60, p = 0.012) (Table 3).

**Table 3:** Plasma biomarker levels in senior airline pilots and office workers Values are presented as mean ± standard deviations. Test statistics for continuous variables are from independent samples t-tests.

Biomarker	Pilots (n = 31)	Office workers (n = 31)	Test statistic	p-value
Phosphorylated tau-181 (pg/mL)	1.36 ± 0.07	1.39 ± 0.08	t = 1.36	0.18
Neurofilament light chain (pg/mL)	7.5 ± 2.4	9.9 ± 3.5	t = 2.60	0.012

Associations between functional and biochemical brain aging markers

**Figure 1:**
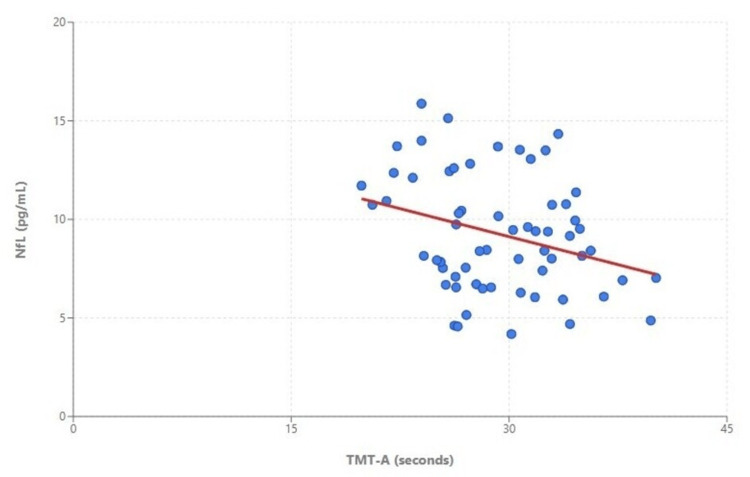
Scattergram and regression line showing a significant inverse relationship between plasma NfL concentrations and Trail Making Test Part A performance in the entire study sample (n = 62; r = –0.30, p = 0.001).

Across the entire sample, higher plasma NfL concentrations were significantly associated with slower processing speed, as evidenced by an inverse correlation with Trail Making Test Part A performance (r = -0.30, p = 0.001; Figure 1). This association remained significant after adjusting for age, years of education, BMI, and blood pressure in a multiple linear regression model (standardized β = -0.27, p = 0.003). No significant correlations were observed between NfL levels and either episodic memory or fine motor performance. Similarly, plasma p-tau181 concentrations were not significantly associated with any cognitive or motor performance measures.

## Discussion

Our investigation revealed three principal findings potentially relevant to aviation policy and cognitive aging research. First, senior CAT pilots demonstrated superior performance on validated measures of processing speed and episodic memory - two cognitive domains particularly vulnerable to age-related decline and critical for flight safety. Specifically, pilots completed the Trail Making Test Part A approximately 15% faster than controls and showed 10-12% better episodic memory performance across both cued recall and auditory verbal learning tasks. Second, pilots exhibited significantly lower plasma concentrations of NfL, a well-established biomarker of neuroaxonal injury [7, 9], while showing no differences in p-tau181 levels. Third, the inverse correlation between NfL concentrations and processing speed across the entire sample provides biological corroboration for the observed functional cognitive differences between groups. Collectively, these findings suggest that the sustained cognitive demands of professional aviation may confer subtle but measurable neuroprotective benefits that can extend into the seventh decade of life.

Our results lend empirical weight to the cognitive reserve hypothesis [14], which posits that sustained engagement in intellectually demanding activities over the lifespan may help mitigate the effects of cognitive aging. Moreover, our findings expand upon accumulating evidence that careers in aviation could offer a certain degree of neuroprotection. In this regard, neuroimaging research conducted by Chen et al. [15] revealed that airline pilots display more robust neurocognitive network activity and increased dynamic connectivity transitions in comparison to non-pilots. Such observations may reflect a higher cognitive flexibility, which could in turn serve as a buffer against age-related physiological decline in cognitive function. From an operational standpoint, these neurobiological observations are supported by findings from Rebok et al. [16], who reported that older pilots do not self-report greater negative changes in abilities compared to younger pilots and, in longitudinal flight simulator assessments, demonstrate less performance decline over time than initially expected. Taylor et al. [5] likewise observed that the oldest pilots experienced less reduction in flight summary scores compared to their younger peers. Notably, these senior pilots even improved their traffic avoidance performance relative to younger pilots, which indicates that the accumulation of aviation-specific expertise and the refinement of operational strategies may serve to counterbalance the anticipated effects of advancing age on performance. Building on this foundation, our investigation represents the first to examine senior CAT pilots using both functional and blood-based brain aging biomarkers, providing unique insight into the biological mechanisms underlying cognitive preservation. Our results revealed substantial processing speed advantages in pilots compared to age-matched office workers, with a notable 4.7-second advantage on Trail Making Test Part A - an effect size that exceeds those typically achieved through cognitive training interventions in older adults [17]. Regarding episodic memory, our findings build upon earlier work by Causse et al. [18], who reported that older pilots with extensive experience displayed stronger spatial working memory than less-experienced counterparts. Notably, our current results extend these observations by showing that memory advantages among senior pilots extend beyond aviation-specific tasks to standardized neuropsychological assessments. This broader applicability points to meaningful transfer effects, supporting cognitive reserve theory by indicating that experience-related benefits may generalize across different memory domains.

The reduced NfL concentrations observed in senior CAT pilots offer biological validation for the subtle yet statistically significant differences in functional brain aging markers found between this professional group and age-matched office workers. In a population-based normal aging cohort of 335 individuals followed for a mean of 5.9 years, Khalil et al. [19] demonstrated that circulating NfL levels become both higher and more variable after 60 years of age, indicating accelerated neuronal injury potentially caused by subclinical pathologies. These findings were corroborated by strong associations between NfL concentrations and brain volume loss, particularly in longitudinal analyses [19]. Compared to age-matched office workers, the senior CAT pilots included in our study showed 24% lower plasma NfL levels, possibly reflecting neuroprotective benefits of sustained cognitive engagement. Notably, across the entire cohort, plasma NfL levels showed an independent inverse correlation with processing speed (Trail Making Test Part A), while no associations were observed with episodic memory or fine motor performance. These findings are consistent with prior work indicating that circulating NfL relates most strongly to processing speed, with weaker or null associations for other domains in healthy older adults; for example, Tang et al. [9] reported significant associations with processing speed but not other domains in 969 community-dwelling men aged 61-73 years. Likewise, our negative results for episodic memory are in accordance with Aschenbrenner et al. [20], who found that NfL predicted decline in attention and global cognition in preclinical Alzheimer’s disease but not memory. Possible explanations for our null findings with respect to memory and motor performance include domain specificity of NfL's cognitive correlates, restricted variance and potential ceiling effects in a high-functioning occupational sample, and limited sample size. Overall, NfL appears independently associated with processing speed but is not a reliable standalone marker for memory or functional mobility in an occupational health context, underscoring the importance of pairing NfL with domain-targeted cognitive measures. Importantly, p-tau181 levels did not differ between the two study groups. Since p-tau181 primarily reflects Alzheimer's disease pathology, this null finding suggests that the cognitive advantages in pilots are likely unrelated to this specific neurodegenerative process. Conversely, the selective reduction in NfL points to broader preservation of neuroaxonal integrity, potentially reflecting enhanced white matter microstructure maintenance.

Upon independent confirmation, our results could significantly impact aviation policy and broaden our understanding of cognitive aging in specialized professions. Accordingly, the superior brain aging resilience in senior CAT pilots nearing mandatory retirement indicates that chronological age may be an inadequate sole criterion for flight fitness determination. A more evidence-based approach incorporating individualized cognitive assessments, cardiovascular risk evaluation, and biomarker profiles could better balance flight safety with optimal utilization of experienced pilots amid current workforce shortages. Future studies should track cognitive and biomarker trajectories longitudinally throughout pilots’ careers, investigate whether similar neuroprotective effects exist in other cognitively demanding professions, and identify which specific aviation elements (e.g., complex decision-making, spatial navigation, or multitasking) may confer the greatest neuroprotective benefits.

Several limitations must be acknowledged when interpreting our preliminary, hypothesis-generating findings. First, the relatively small sample size may have limited statistical power to identify more subtle associations, particularly between p-tau181 and cognitive measures. Second, our sample consisted exclusively of male participants, reflecting the historical gender composition of senior commercial aviation. Given established sex differences in cognitive aging and biomarker profiles, these findings cannot be generalized to female pilots. Third, although we controlled for education, BMI, and blood pressure, unmeasured lifestyle factors may have contributed to the observed group differences. In particular, we did not directly assess smoking, alcohol consumption, habitual physical activity, global health status, or sleep quality, each of which can influence brain-aging biomarkers and cognitive/operational performance and may partially account for between-group differences. Finally, our cross-sectional design cannot determine whether aviation careers confer neuroprotective benefits or whether cognitively resilient individuals self-select into and persist in CAT piloting.

## Conclusions

This study provides preliminary evidence that senior CAT pilots exhibit cognitive performances and a biochemical marker profile associated with healthier brain aging. These results challenge the scientific basis of current age-based mandatory retirement policies and support transitioning toward competency-based assessments that better balance flight safety with workforce needs. As the aviation industry confronts severe pilot shortages amid ongoing demographic shifts, our findings indicate that experienced senior pilots may represent an underutilized resource whose cognitive capabilities can substantially exceed those predicted by chronological age alone. Future longitudinal studies tracking pilots throughout their careers will be essential for developing evidence-based policies that optimize both aviation safety and workforce sustainability in an aging society.
